# Fermented So-Cheong-Ryong-Tang (FCY) induces apoptosis via the activation of caspases and the regulation of MAPK signaling pathways in cancer cells

**DOI:** 10.1186/s12906-015-0821-2

**Published:** 2015-09-24

**Authors:** Nam-Hui Yim, Aeyung Kim, Young Pil Jung, Taesoo Kim, Choong Je Ma, Jin Yeul Ma

**Affiliations:** KM-Application Center, Korea Institute of Oriental Medicine, 1672, Yuseongdaero, Yuseong, Daejeon, 305-811 Republic of Korea; Department of Biomaterials Engineering, Division of Bioscience and Biotechnology, Kangwon National University, Chuncheon, 200-701 South Korea

**Keywords:** Socheongryong-tang (CY), Fermentation, Apoptosis, Caspase activity, Mitogen-activated protein kinases (MAPKs), Xenograft assay

## Abstract

**Background:**

So-Cheong-Ryong-Tang (CY), a traditional herbal formula, mainly has been shown to possess allergic rhinitis and asthma for hundreds of years in Asian countries. Although this medicine has been attracted Asian scientists with investigating mechanisms of action against inflammatory-related diseases, there is a little available information on the anti-cancer effect of CY, especially on the fermented form (FCY). In this study, we explored the chemopreventive/chemotherapeutic efficacy of FCY against cancer cells and proved the efficacy of FCY through performing in vivo xenograft assay.

**Methods:**

CY was fermented with bacteria and lyophilized. For analysis of the constituents of CY and FCY, high performance liquid chromatography (HPLC)-DAD system was performed. To detect the anti-cancer effect of FCY, cell viability assay, caspase activity assay, cell cycle analysis, and Western blot analysis were performed in AGS human gastric cancer cells. The inhibitory effects of tumor growth by CY and FCY were evaluated in athymic nude mice inoculated with HCT116 human colon cancer cells.

**Results:**

As a result of analyzing the 11components present in CY and FCY, the contents of ephedrine HCl, glycyrrhizin, gingerol, schisandrin, and gomisin A were respectively increased by fermentation in FCY. The treatment of CY or FCY inhibited the viability of AGS cells, interestingly, the inhibition of cancer cell growth was enhanced by fermentation of CY. FCY induced the apoptosis through activating the caspase-3, −8, and −9. Additionally, FCY regulated the activation of mitogen-activated protein kinases (MAPKs) including extracellular signal-regulated kinase (ERK), p38, and c-Jun NH2-terminal kinase (JNK). In vivo xenografts, administration of FCY significantly inhibited the tumor formation, and improved the anti-tumor effect compared to that of CY in athymic nude mice.

**Conclusions:**

FCY indicated significant anti-cancer effects, and its efficacy against tumor formation was improved than that of CY, therefore, FCY might be used for applications of traditional medicine against cancer in modern complementary and alternative therapeutics.

**Graphical Abstract:**
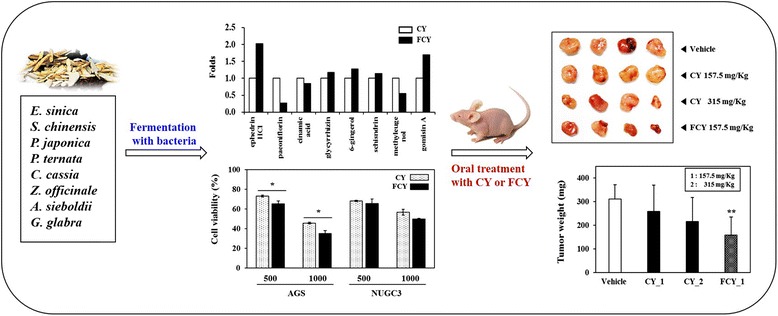
ᅟ

## Background

Cancer, particularly in its advanced stages, is a multifactorial disease that demands treatments that target multiple cellular pathways; several options are available for this purpose. However, chemotherapy with cytotoxic anti-cancer drugs is associated with significant side effects and offers few survival benefits for patients with advanced malignancies. Thus, some patients and clinicians consider the use of alternative medicines as another treatment option [1, 2].

Formulated herbal cocktails target multiple cellular pathways to correct the dysregulated cellular functions that are associated with the various stages of cancer development [3]. However, herbal remedies have yet to be fully integrated into mainstream medicine due to a lack of experimental and clinical studies of their safety, efficacy, and underlying pharmacological mechanisms. Therefore, multi-herbal cocktails must be pre-clinically evaluated to accurately compare traditional herbal medicines with modern therapeutics [4].

The traditional Oriental medicine, So-Cheong-Ryong-Tang (CY), which is also known as Xiao-Qing-Long Tang in China and Sho-Seiryu-To in Japan, is an herbal mixture that has for hundreds of years been used to treat diseases such as allergic rhinitis and asthma in Asian countries [5, 6]. Recent evidence indicates that the specific bioactivities of CY include the modulation of gastrointestinal motility [7], the influencing of gene expression in a rodent model of chronic obstructive pulmonary disease [8], the regulation cytokines and chemokines on allergic airway inflammation [5], and the induction of apoptosis in human lung cancer cells [9]. Additionally, previous studies from our laboratory have suggested that CY has protective effects against hydrogen peroxide-induced hepatotoxicity [10]. CY is an aqueous polyherbal formulation that contains eight herbs: *Ephedra sinica*, *Schisandra chinensis*, *Paeonia japonica*, *Pinellia ternata*, *Cinnamomum cassia*, *Zingiber officinale*, *Asarum sieboldii*, and *Glycyrrhiza glabra*. These constituent herbs and their components have been reported to have anti-carcinogenic effects. In particular, *E. sinica* [11], *S. chinensis* [12], *C. cassia* [13], and *G. glabra* [14] inhibit tumor growth and metastasis, whereas *P. ternate* [15] and *Z. officinale* [16] induce apoptosis in cancer cells. These findings suggest that CY may have an inhibitory effect on cancer cell proliferation and tumor formation. On the basis of these reports, in present study, CY was treated to several human cancer cells including AGS (gastric), HeLa (cervical), and PC3 (prostate) cells. Among them, the inhibition of CY against cancer cell viability showed the strong sensitivity in AGS cells.

Tumorigenesis is caused by uncontrolled cell growth resulting from DNA damage, the mutation of functional genes, a dysregulation of the cell cycle, or the loss of apoptotic functions [17]. Therefore, regulating the induction of apoptosis via the modulation of signaling pathway-related cell growth and survival is a common and major target for cancer therapies [18]. Mitogen-activated protein kinase (MAPK) signals such as extracellular signal-regulated kinases (ERK), p38 kinases, and c-Jun N-terminal kinases (JNK) play important roles in cell death and survival. ERK activation may be induced by conditions of stress caused by some agents or oxidant injury and plays a major role in the regulation of cell growth and differentiation. JNK and p38 are activated in response to several stress signals, including tumor necrosis factor and hyperosmotic conditions, and are associated with induction of apoptosis [19, 20].

Recently, many researchers have adopted the use of bioconversion techniques, such as microbial transformation or bacterial fermentation, to develop natural materials with strengthened efficacy and/or targeted function. For these purposes, research on the beneficial effects of bioconversion using microbes has aimed to elucidate the mechanisms of action underlying the benefits of these natural materials and to analyze the herbal constituents that exhibit the greatest efficacy [21–23]. In this respect, our laboratory has previously prepared fermented CY (FCY), analyzed its constituent compounds, and reported its acute toxicity and safety using imprinting control region (ICR) mice [24]. An experimental study from Japan investigated whether the efficacy of CY was affected by a probiotic product, and concluded that CY-induced anti-allergic effects were enhanced by the probiotic [25]. In the present study, the anti-carcinogenic effects of FCY were investigated in vitro using gastric cancer cells and in vivo using colon cancer cells in preclinical experiments. The present findings indicate that FCY induced apoptosis in these cancer cells via the regulation of MAPK signaling cascades.

## Methods

### Materials and reagents

Dulbecco’s modified Eagle’s medium (DMEM) and RPMI-1640 were obtained from Lonza (Walkersville, MD, USA). Fetal bovine serum (FBS), penicillin and streptomycin, and peroxidase-conjugated secondary antibodies were purchased from Hyclone (Logan, UT, USA). Propidium iodide (PI) and 3-[4, 5-dimethylthiazol-2-ly]-2, 5-diphenyl-tetrazolium bromide (MTT) were purchased from Sigma Chemical Co. (St. Louis, MO, USA). Caspase-Glo 3/7, −8 and −9 assay kits were purchased from Promega (Madison, WI, USA). GAPDH, caspase-3, caspase-8, caspase-9, PARP, cyclin D1, cyclin B1, cyclin E1, p21, p27, ERK, phospho-ERK, p38, phospho-p38, JNK, and phospho-JNK were purchased from Cell Signal Technology, Inc. (Boston, MA, USA). For reference standards, ephedrine HCl, glycyrrhizin, 6-gingerol, gomisin A, gomisin N, paeoniflorin, and schisandrin were purchased from the Korea Food and Drug Administration (Cheongwon, Korea). Cinnamaldehyde, cinnamic acid, homogentisic acid and methyl engenol were purchased from Sigma Chemical Co. (St. Louis, MO, USA). HPLC grade solutions including water, acetonitrile and methanol were purchased from J.T. Baker (Austin, TX, USA) and Trifluoroacetic acid for analysis reagent was purchased from DAE JUNG Chemical & Materials Co. (Siheung, Korea).

### Herb materials and preparation of fermented CY

CY was composed of 8 medicinal herbs; their constitution ratio is shown in Table 1. The 8 herbs were purchased from the Korea Medicine Herbs Association (Yeongcheon, Korea). The herbal mixture was extracted by heating in water of 8–10-fold the herb weight for 3 h at 115 °C on Cosmos–600 extractor (Incheon, Korea). After boiling, the extract was filtered out using standard testing sieves (pore size, 150 μm) (Retsch, Haan, Germany) and prepared in the form of powder by freeze-drying. The CY was incubated with Lactobacilluses (1–5 × 10^8^ CFU/mL) obtained from the KFRI (Korea Food Research Institute, Sungnam, Korea) to prepare fermented CY. Before use, the bacterial strain was incubated in 50 mL of MRS broth (Difco ^TM^ Lactobacilli MRS Broth, Becton Dickinson, Franklin Lakes, NJ) at 37 °C overnight. The fermented CY (FCY) by Lactobacillus at 37 °C for 48 h was filtered with a 60 μm naylon filter (Millipore, Billerica, MA), lyophilized, and stored −20 °C before use. The voucher specimen of FCY (Registration no. CY442) was deposited in the herbarium of KM-Based Herbal Drug Development Group, Korea Institute of Oriental Medicine.

**Table 1:** Composition of the So-Cheong-Ryong-Tang (CY) preparation

No.	Scientific name	Part used	Amount used (g)
1	*Ephedra sinica*	Radix	6
2	*Schisandra chinensis*	Fructus	6
3	*Paeonia lactiflora*	Radix	6
4	*Pinellia ternate*	Rhizoma	6
5	*Cinnamomum cassia*	Radix	4
6	*Zingiber officinale* Rosc.	Radix	4
7	*Asiasarum heterotropoides*	Rhizoma	4
8	*Glycyrrhiza glabra* Fisch.	Radix	4
Total amount			40

### Characterization of phytochemicals

To determine phytochemical profile and quantify chemical components of CY and FCY, HPLC analysis was performed using the method reported previously with a Dionex Ultimate 3000 HPLC system (Dionex, Germany) and LUNA C18 column (250 × 4.60 mm i.d., 5 μm) [24]. The mobile phase was consisted of acetonitrile (A) and 0.1 % (v/v) trifluoroacetic acid (B). The line program was well optimized and conducted as follow: 15 % A at 0–5 min; 15–40 % A at 5–20 min; 40–45 % A at 20–30 min; 45–43 % A at 30–40 min; and 43–100 % A at 40–60 min at a flow rate of 1.0 mL/min. The ultraviolet (UV) spectrum was recorded between 190 and 400 nm. The injection volume was set at 20 μL. Components were identified via comparision of their retention times to those of authentic standards under identifical analysis conditions and UV spectra with an in-house PDA-library. The standard stock solutions of the eleven reference standards were prepared by dissolving in methanol to a final concentration of 140 μg/mL for ephedrine HCl, 240 μg/mL for paeoniflorin, 200 μg/mL for glycyrrhizin, 240 μg/mL for schidandrin, 210 μg/mL for gomisin A, 240 μg/mL for gomisin N, 265 μg/mL for 6-gingerol, 350 μg/mL for homogentisic acid, 260 μg/mL for methyl engenol, 163.83 μg/mL for cinnamaldehyde, and 130 μg/mL for cinnamic acid, respectively. CY and FCY powder were weighed and dissolved with deionized water at a concentration of 20 mg/mL. Prior to analysis, the extracted solution was filtered through 0.45 μm filter and maintained at 4 °C.

### Cell culture

Various human cancer cell lines, obtained from the Korean Cell Line Bank (KCLB, Seoul, Korea) and American Type Culture Collection (ATCC, Rockville, MD), were cultured in DMEM and RPMI-1640 supplemented with 10 % FBS. All media contained 100 U/mL penicillin G and 100 μg/mL streptomycin. Cells were incubated in a humidified 5 % CO_2_ atmosphere at 37 °C.

### Cell viability assay

Cells (4 × 10^3^ or 5 × 10^3^ per well) were inoculated in a 96-well plate and treated with CY or FCY for 24 or 48 h. After incubation, cell viability was determined using MTT colorimetric assay based on the reduction of tetrazolium salt to its insoluble formazan. For inhibitor study, cells were pretreated inhibitors for 1 h and treated with FCY for 48 h. After incubation, cell viability was determined using MTT assay.

### Caspase activity assay

To determine caspase-3/7, −8, and −9 activities, cells were seeded at a density of 1 × 10^4^ cells/well in a 96-well plate and treated with CY and FCY for 24 h. Caspase activity was measured in triplicate by using a respective Caspase-Glo 3/7, −8 and −9 assay kits according to the manufacturer’s instructions. Culture medium was used as a blank control and luminescence was measured using an MLX microtiter luminometer (Dynex Technologies Inc., Chantilly, VA).

### Cell cycle analysis

Cells were seeded at a density of 1 × 10^5^ /mL and treated with 500 and 1000 μg/mL FCY for 24 h. The PI staining for cell cycle analysis was performed as described previously (Yim et al., 2011). DNA contents of the stained cells were analyzed by FACSCalibur flow cytometry using CellQuest software (Becton–Dickinson, Franklin Lakes, NJ).

### Detection of YO-PRO-1 uptake

For the measurement of apoptosis, cells treated with 500 and 1000 μg/mL FCY for 24 h were incubated with apoptosis-specific dye YO-PRO-1 (1 μM, Molecular Probes, Eugene, OR) at 4 °C for 30 min in the dark. YO-PRO-1 uptake was directly determined with FACS Calibur flow cytometry without washing or fixation and analyzed using FCS express software.

### Western blot analysis

The cell lysates treated with CY or FCY for western blot anlaysis were prepared as described previously [26]. The same amount of protein for each sample was electrophoresed and transferred onto a polyvinylidene difluoride (PVDF) membrane (Millipore, Billerica, MA). Proteins were detected using primary antibodies specific for GAPDH, caspase-3, caspase-8, caspase-9, PARP, cyclin D1, cyclin B1, cyclin E1, p21, p27, ERK, phospho-ERK, p38, phospho-p38, JNK, and phospho-JNK. This was followed by incubation with HRP-conjugated secondary antibodies for 1 h at room temperature. The specific protein was detected using the enhanced chemiluminescence imaging system (CoreBio, Seoul, Korea).

### Animals and tumor xenografts

Female mice (Athymic nu/nu, 8 weeks, 25–29 g; NARA Bio, Seoul, Korea) were acclimated under conditions of constant temperature (24 ± 1 °C) and humidity (55 ± 15 %) with 12-h light/dark cycle for 1 week. Mice were injected subcutaneously with 4 × 10^6^ HCT116 cells/100 μL harvested and suspended in DMEM medium without FBS. On day 5 postinoculation, mice were randomized into groups (*n* = 4 per group) and daily administrated with saline (control), CY (157.5 or 315 mg/Kg) or FCY (157.5 mg/Kg) in a volume of 100 μL for 14 days. Tumor size was monitored using electronic caliper on every alternate day and tumor volume was calculated according to the following formula: tumor volume = length × width × width/2. The experiment was terminated at the end of 15 days when the vehicle-treated animals had large tumors, and tumor was removed for the measurement of tumor weights. The animal experimental procedures were approved by Korea Institute of Oriental Medicine Care and Use Committee with a reference number of #13–095 and performed in accordance with the Korea Institute of Oriental Medicine Care Committee Guide lines.

### Statistical analysis

Data are presented as means ± SD. Student’s *t*-test was employed to assess the statistical significance of differences between the control and CY- or FCY-treated groups. Values of *p* <0.05 and <0.01 were considered to indicate statistical significance.

## Results

### Phytochemical characterization of CY and FCY

**Fig. 1:**
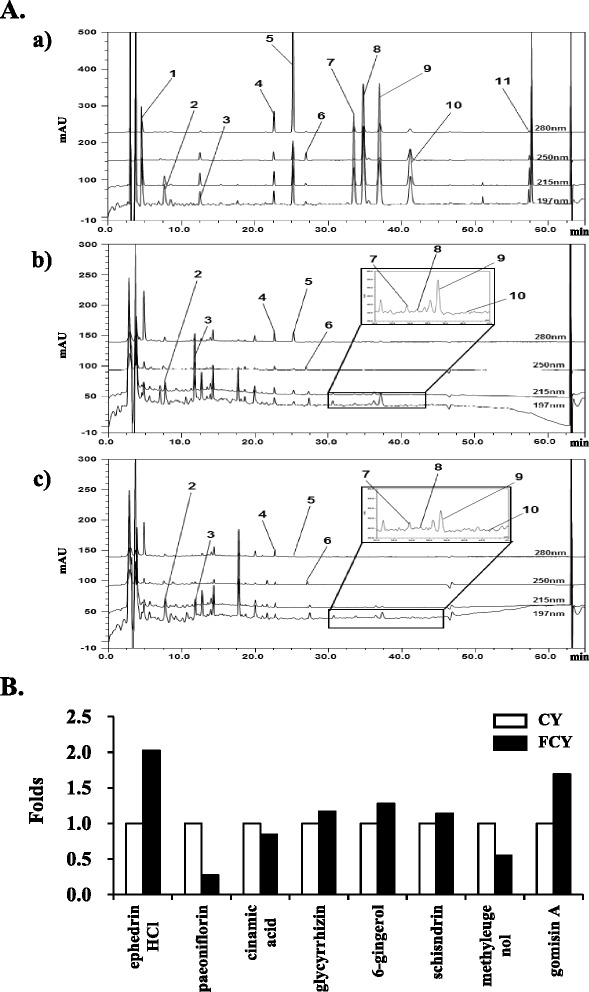
HPLC-DAD analysis of CY and FCY. **a** Determination of eleven standard components in CY and FCY. (*a*) Mixed standards, (*b*) CY, and (*c*) FCY. **b** Identification of changed components by bacterial fermentation in FCY compared to CY

The present study utilized high-performance liquid chromatography (HPLC) to analyze the 11 primary components present in CY and FCY using conditions reported previously [24]. Based on the stability and higher maximum absorption rates of the major components at baseline, the components were selected using four ultraviolet (UV) wavelengths: 197 nm for homogentisic acid, ephedrine HCl, paeoniflorin, 6-gingerol, and methyl eugenol, 215 nm for schisandrin, gomisin A, and gomisin N, 250 nm for glycyrrhizin, and 280 nm for cinnamic acid and cinnamic aldehyde (Fig. 1a). The contents of the 11 components in CY and FCY were quantified using UV and mass spectroscopy (MS) spectra and their retention times were compared to standards using methods described previously (Table 2). The quantitative analysis data are shown in Fig. 1b. The contents of ephedrine HCl (+102.48), glycyrrhizin (+16.98), 6-gingerol (28.03), schisandrin (+13.98), and gomisin A (+69.23) in FCY were higher than those in CY (100). In particular, the levels of ephedrine HCl and gomisin A were markedly higher in FCY compared to CY. In contrast, the contents of paeoniflorin (−72.53), cinnamic acid (−15.22), and methyl eugenol (−44.90) in FCY were lower than those in CY (100). The HPLC analysis detected low levels of homogentisic acid, cinnamic aldehyde, and gomisin N; however, their amounts were below the limits of quantification (LOQ) and were recorded as not detected (nd).

**Table 2:** Characterization of the standard compounds in CY and FCY using HPLC

	Compound name	Classification^a^	tR (min)			Wavelength (nm)
			Standard	CY	FCY	
1	Homogentisic acid	4	4.75	n.d.	n.d.	197
2	Ephedrine HCl	1	7.11	7.08	7.30	197
3	Paeoniflorin	3	11.85	11.83	11.83	197
4	Cinnamic acid	5	21.42	21.53	22.69	280
5	Cinnamic aldehyde	5	24.58	24.59	25.27	280
6	Glycyrrhizin	8	26.35	26.45	26.93	250
7	6-Gingerol	6	33.71	33.61	33.61	197
8	Schisandrin	2	34.83	34.71	34.71	215
9	Methyl eugenol	7	37.80	37.70	37.80	197
10	Gomisin A	2	42.32	42.40	42.40	215
11	Gomisin N	2	59.29	n.d.	n.d.	215

^a^1, *Ephedra sinica*; 2, *Schisandra chinensis*; 3, *Paeonia lactiflora*; 4, *Pinellia ternate*; 5, *Cinnamomum cassia*; 6, *Zingiber officinale* Rosc.; 7, *Asiasarum heterotropoides*; 8, *Glycyrrhiza glabra* Fisch

### Inhibition of cell growth by CY and FCY in human gastric cancer cells

**Fig. 2:**
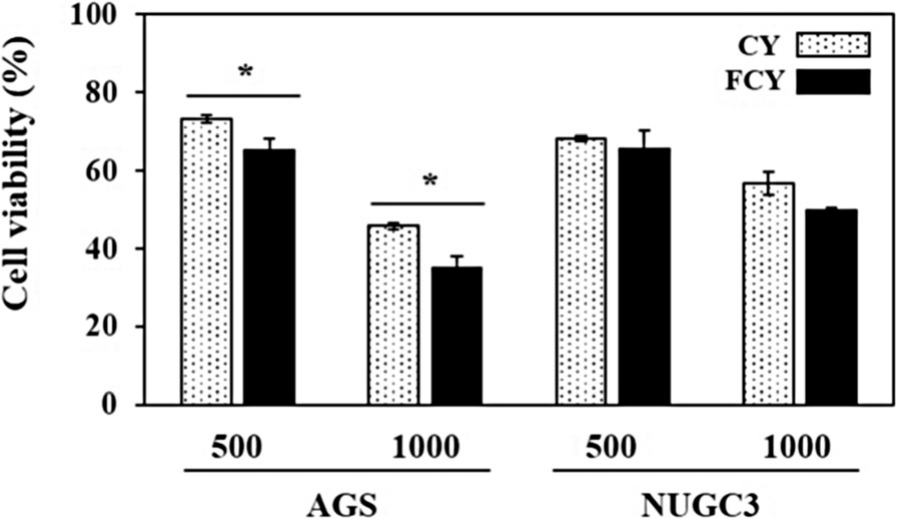
Inhibition of human gastric cancer cell viability by CY or FCY. AGS and NUGC cells were treated with 500 and 1000 μg/mL for 48 h. Cell viability was determined by MTT assay and the results are expressed as the percentages of viable cells compared to untreated cells. The data are shown as the means ± SD of three independent experiments

To further characterize the inhibitory actions of CY and FCY on cancer cells, the abilities of these compounds to suppress the growth of two gastric cancer cell lines (AGS and NUGC-3) were evaluated. Treatment with CY (500 and 1000 μg/mL) for 48 h reduced the viability of AGS cells by 27 and 55 %, respectively, and treatment with FCY (500 and 1000 μg/mL) inhibited AGS cell growth by approximately 35 and 65 %, respectively (Fig. 2). Additionally, the growth inhibitory effect of FCY on AGS cells was significantly greater than that of CY. Similarly, treatment with either CY or FCY (500 μg/mL) reduced the viability of NUGC-3 cells; however, this difference was not significant although CY and FCY (1000 μg/mL) inhibited NUGC-3 cell growth. These findings indicate that CY and its fermented formulation, FCY, exert cancer-specific cytotoxic effects on AGS cells and that FCY has greater anti-carcinogenic efficacy than CY. Thus, the present study focused on AGS cells for subsequent tests involving FCY.

### Induction of apoptosis by FCY via the activation of caspases in AGS cells

**Fig. 3:**
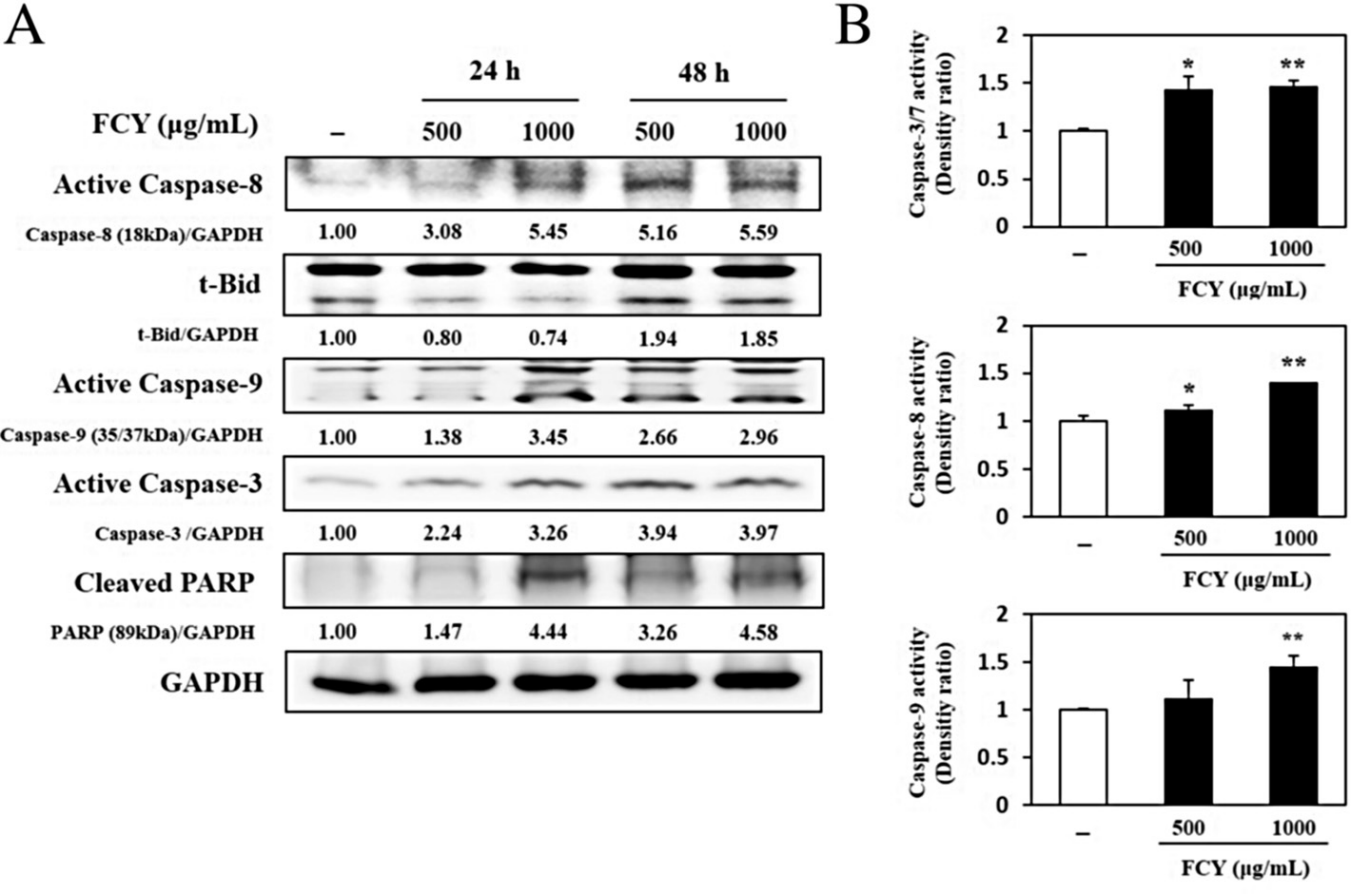
Induction of apoptosis by FCY in AGS cells. **a** Effects of FCY on the expressions of caspase-3, −8, and −9, and PARP cleavage. The cells were exposed to 500 and 1000 μg/mL FCY for 24 and 48 h and protein levels were determined by Western blot analyses. The band intensity was calculated and compared to untreated cells using ImageJ after normalization relative to GAPDH expression. **b** The relative luminescence indicated the dose-dependent activations of caspase-3/7, −8, and −9 that were induced by 48 h of treatment with the indicated dose of FCY. The data are shown as the means ± SD of three independent experiments. ^*^ *P* < 0.05 and ^**^ *P* < 0.01 versus untreated control cells

To determine whether the cell death induced by FCY was related to apoptosis, the expressions of pro- and anti-apoptosis proteins in the AGS cells were assessed using Western blot analyses. The expression levels of apoptosis-related proteins were clearly influenced by FCY treatment (Fig. 3a). Compared to untreated cells, treatment with FCY (500 and 1000 μg/mL) for 48 h induced the activations of caspase-3, −8, and, −9 in AGS cells by more than threefold. FCY treatment (1000 μg/mL; 48 h) also increased the levels of truncated bh3-interacting domain death agonist (t-Bid) and cleaved poly (ADP-ribose) polymerase (PARP) by approximately two- and four-fold.

To confirm that FCY-induced apoptosis in AGS cells required caspase activation, a caspase activity assay was performed (Fig. 3b). FCY (500 and 1000 μg/mL) increased caspase activity after 48 h of treatment, and caspase-3/7, −8, and −9 activities increased by approximately 46, 39 and 45 %, respectively, after treatment with 1000 μg/mL FCY compared to untreated cells. These data support the hypothesis that the anti-carcinogenic effects of FCY in AGS cells were related to apoptotic cell death resulting from the activation of caspases.

### Effect of FCY on cell cycle progression in AGS cells

**Fig. 4:**
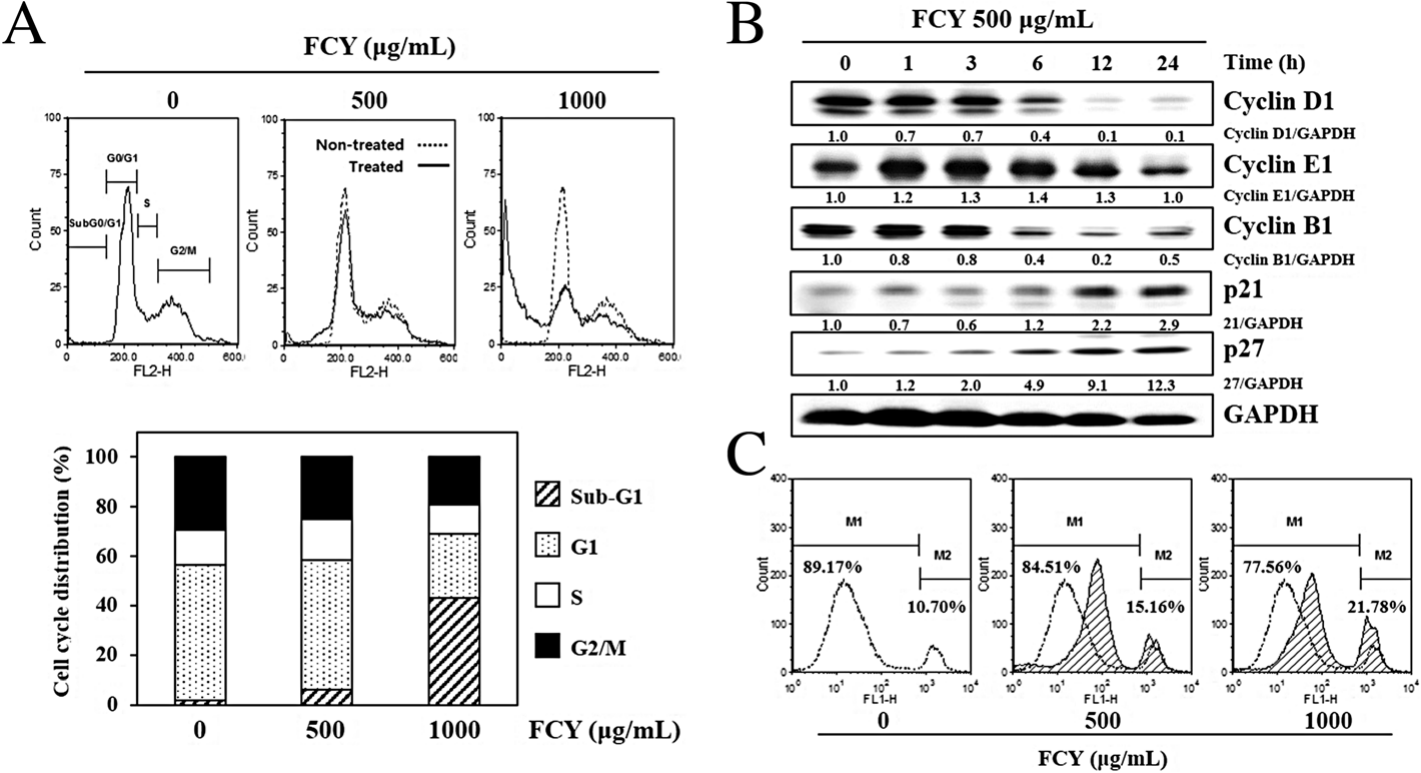
Effects of FCY on cell cycle progression in AGS cells. **a** The cells were treated with 500 or 1000 μg/mL of FCY for 48 h, fixed with pre-chilled 70 % ethanol, stained with propidium iodide solution and then subjected to flow cytometry for determination of cell cycle distribution. **b** The levels of cell cycle regulatory proteins in cells treated with FCY for 24 h were examined by Western blot analyses. **c** After incubation with 500 or 1000 μg/mL FCY, apoptosis was assessed using YO-PRO-1 staining by flow cytometry. The percentage of the M2 population depicts apoptosis, which increased in conjunction with the dose. The data are representative of three independent experiments

After treatment with FCY (500 and 1000 μg/mL) for 48 h, the AGS cells were stained with propidium iodide (PI) and their cell cycle progression was assessed using flow cytometry. FCY increased the number of cells in the sub-G1 peak in a dose-dependent manner, which is consistent with the induction of cell death (Fig. 4a). After 48 h of treatment with FCY (500 and 1000 μg/mL), 6.25 and 43.04 % of the cells (three- and four-fold increases), respectively, had accumulated in the sub-G1 phase compared to untreated cells (1.92 %). Additionally, 52.11 and 26.04 % of cells treated with FCY (500 and 1000 μg/mL, respectively) had accumulated in the G1 phase after 48 h. Following treatment with FCY (500 and 1000 μg/mL), 25.31 and 19.3 % of cells accumulated in the G2/M phase, respectively, lower than the proportion of untreated cells (29.45 %).

Based on these data, the present study then investigated whether the expressions of cell cycle-regulating proteins were affected by FCY treatment. FCY altered the expressions of proteins associated with the G1 and G2/M phase progressions (Fig. 4b). Specifically, the expressions of p21 and p27 increased in conjunction with a reduction in cyclin D1 levels in a time-dependent manner. The levels of cyclin B1, which regulates the G2/M phase, were also decreased following treatment with FCY, but cyclin E1 levels were unaffected.

Additionally, apoptotic cell death following FCY treatment was assessed using YO-PRO-1 staining and flow cytometry (Fig. 4c). Compared to untreated cells, the percentage of cells distributed in the M2 population, which signifies apoptosis, increased by approximately 5 and 11 %, respectively, following treatment with 500 and 1000 μg/mL FCY. These data indicate that the effect of FCY on the cell cycle suppresses DNA synthesis and growth in AGS cells, which are related to apoptosis.

### Identification of MAPK phosphorylation by FCY for anti-cancer activity in AGS cells

**Fig. 5:**
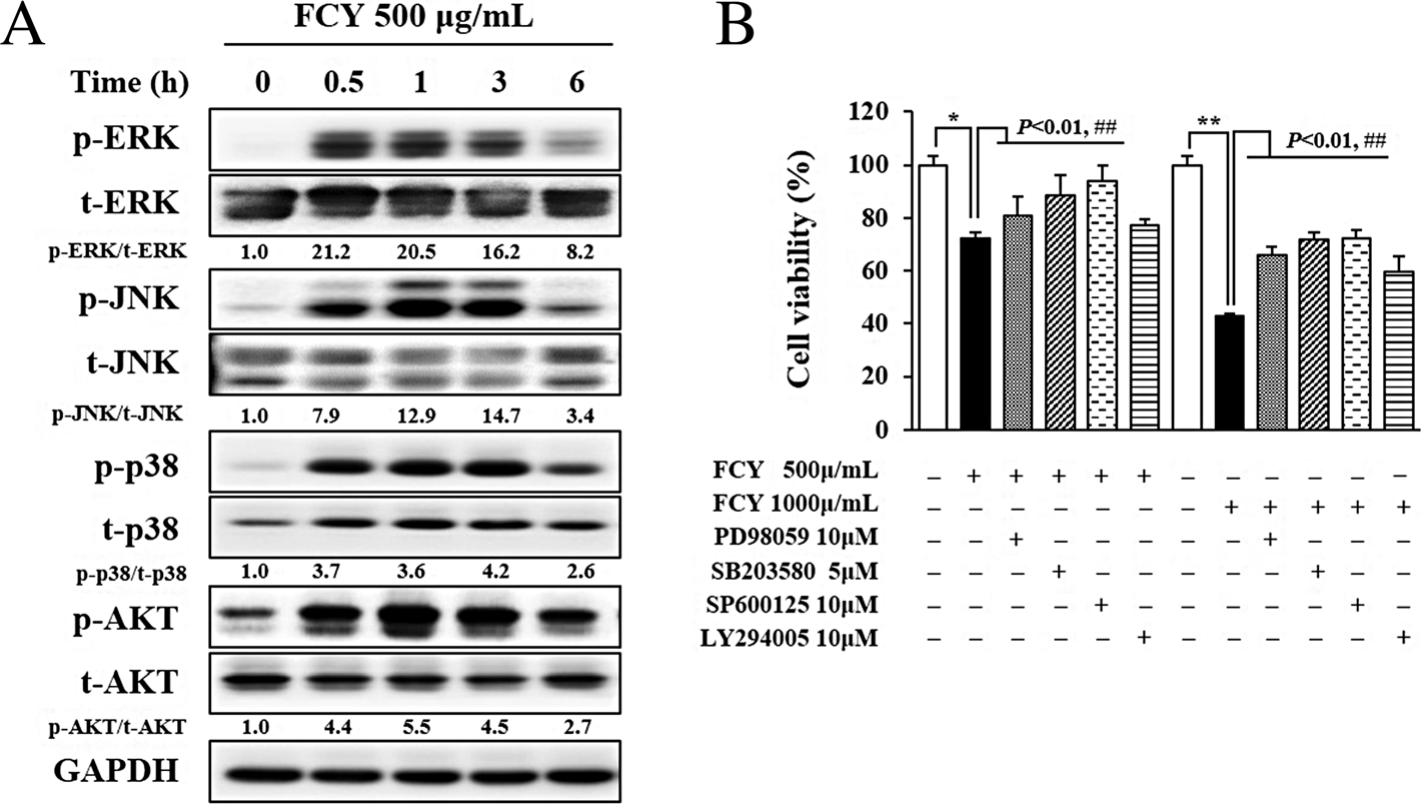
Identification of the relationship between MAPK activation and the anti-proliferative effects of FCY on AGS cells. **a** Cells were prepared after treatment with 500 μg/mL FCY for 0.5, 1, 3, and 6 h, and then subjected to Western blot analyses to determine the levels of MAPK proteins, including ERK, p38, and JNK (or AKT) and their phosphorylated forms. **b** Investigation of the anti-proliferative effects of FCY for 48 h using the MAPK cascade inhibitors PD98059 (10 μM), SB203580 (5 μM), and SP600125 (10 μM), and the PI3K inhibitor LY294005 (10 μM). Cell viability was determined by MTT assay. The results show the means ± SD of three independent experiments. ^*^ *P* < 0.05 and ^**^ *P* < 0.01 versus untreated control cells and ^##^ *P* < 0.01 versus cells treated with FCY only

To investigate the relationship between the regulation of MAPK pathways and the inhibition of cancer cell proliferation following FCY treatment, the FCY-induced phosphorylation of MAPK proteins was analyzed by Western blotting. The phosphorylation of MAPK cascades, including ERK, p38, and JNK, increased after 30 min of treatment with FCY (500 μg/mL), which was sustained for 6 h in AGS cells (Fig. 5a). In contrast, AKT phosphorylation was transiently upregulated following 6 h of exposure to FCY. To further investigate the regulation of these signaling pathways by FCY, AGS cells were pretreated with the MAPK inhibitors PD98059 (inhibits ERK1/2), SB203580 (inhibits p38), and SP600125 (inhibits JNK) and then treated with FCY (500 or 1000 μg/mL) for 48 h (Fig. 5b). Each of the MAPK inhibitors significantly diminished the anti-proliferative effects of FCY in AGS cells, especially SP203580, which reduced cell death by 40 % compared to 1000 μg/mL FCY. In contrast, the anti-proliferative effects of FCY were not enhanced by LY294002. Taken together, these data suggest that FCY exerts anti-proliferative effects in AGS cells via the modulation of MAPK signaling pathways, which, in turn, results in the induction of apoptosis.

### Inhibition of tumorigenic growth of cancer cells by FCY administration in xenograft model

**Fig. 6:**
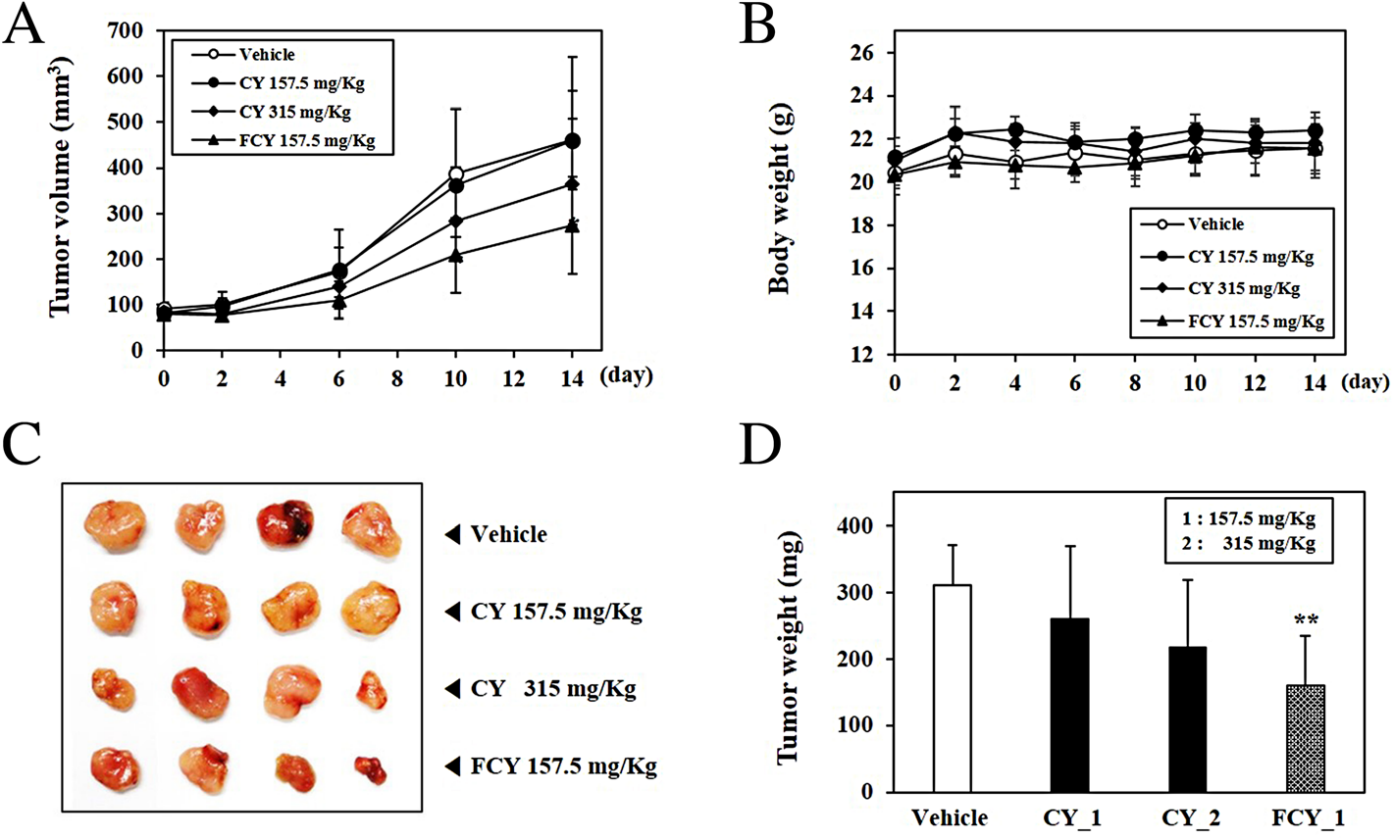
Inhibitory effects of FCY on in vivo tumor growth in a xenograft model. (**a**) The cells were injected into athymic nude mice and, 5 days after tumor implantation, the mice were treated daily with saline (vehicle), CY (157.5 mg/kg or 315 mg/kg), or FCY (157.5 mg/kg) for 14 days. Treatment was terminated after 15 days. (**b**) Changes in body weight during the administration of CY or FCY and tumor growth inhibition by (**c**) CY or (**d**) FCY. Comparison of anti-tumor activity between vehicle and herbal formulations (CY and FCY), according to representative tumor images and tumor weights. Data are shown as means ± SD, ^*^ *P* < 0.05 versus vehicle

To confirm these observations, the inhibitory effects of CY and FCY on tumor growth were assessed in athymic nude mice injected with HCT116 human colon cancer cells. Mice harboring xenograft tumors were treated orally with either vehicle (control), CY-1 (157.5 mg/kg/day), CY-2 (315 mg/kg/day), or FCY-1 (157.5 mg/kg/day). The CY and FCY doses were based on the amounts used in human adults (9.456 g/60 kg/day) and the yield of the powdered extraction (23.64 %). After 6 days of herb administration, there was an arrest in the growth of the xenografts treated with CY-2 and FCY as well as reductions in tumor size at the end of the experiment on day 15 (Fig. 6a). Additionally, none of the orally administered herbal formulations resulted in adverse side effects, such as a loss of body weight or skin ulcers (Fig. 6b). Treatment with FCY led to a significant inhibition of 48.6 % in tumor growth compared to vehicle. Although the administration of CY-1 and CY-2 to mice harboring xenograft tumors reduced tumor growth by approximately 16 and 30 %, respectively, these changes were not statistically significant (Fig. 6c and d). These results provide strong evidence for the anti-carcinogenic effects of FCY in vivo.

## Discussion

Traditional medicine in Asian countries commonly involves the combination of herbs to create multi-herbal formulas for the treatment of various diseases. The use of such formulas has been scientifically verified as a complementary or alternative therapy for the treatment of cancer [27, 28]. CY is an important oriental medicine that has traditionally been used to modulate autoimmune diseases such as allergic rhinitis and asthma [5, 6, 29]. In the present study, CY and FCY inhibited the growth of human gastric cancer cells and cancer cell death was particularly enhanced following treatment with FCY.

Based on these preliminary observations, the molecular mechanisms underlying the anti-carcinogenic effects of FCY were assessed in AGS cells. Using Western blot analyses, FCY was shown to influence the expression levels of pro- and anti-apoptotic proteins; these results were confirmed by caspase activity assays. The caspase cascades are divided into two major pathways: an extrinsic pathway containing caspase-8 and −10 that is initiated by the ligand-mediated activation of cell surface death receptors, and an intrinsic pathway containing caspase-9 that is activated by intracellular signals from the mitochondria. The activation of initiator caspases such as caspase-8, −9, and −10 by pro-apoptotic signals leads to the downstream activation of effector caspases such as caspase-3, −6, and −7 [30, 31]. In the present study, the FCY-stimulated activation of caspase-8 resulted in an increased level of t-Bid, which is typical of the initiation of the extrinsic apoptosis pathway. In turn, t-Bid influenced the activations of caspase-9 and −3 and the cleavage of PARP. Therefore, the present data suggest that FCY induced apoptosis via caspase-dependent pathway in AGS cells.

The anti-proliferative effects of FCY were also identified by assessing changes in cell cycle progression. Cell cycle checkpoints are important control mechanisms that ensure the proper execution of cell cycle events. In eukaryotes, the cell cycle progression is divided into three phases: the G1/S phase, which involves DNA synthesis and replication, the G2/M phase, which is associated with mitosis and complete cell division, and the sub-G1 phase, in which the cells have left the cycle and stopped dividing [32–34]. During FCY-induced apoptosis in AGS cells, the expressions of G1/S and G2/M checkpoint proteins, including cyclin D1, p21, p27, and cyclin B1, were affected by FCY treatment. Additionally, the cell population in the sub-G1 phase increased in a concentration-dependent manner following treatment with FCY. Thus, the present data suggest that FCY inhibited the proliferation of cancer cells via the disruption of cell cycle progression.

MAPKs regulate cellular processes such as the proliferation, differentiation, and apoptosis of cells [35]. In particular, the pharmacological modulation of MAPK signals influences the apoptotic response to anti-tumor agents [19]. Furthermore, the MAPK signaling pathways regulate diverse cellular programs by relaying extracellular signals to intracellular responses [20]. Hwang et al. demonstrated that MAPK proteins are involved in the CY-induced modulation of pacemaker potentials in the interstitial cells of Cajal (ICCs) [7]. Similarly, the present study found that FCY treatment activated the ERK, p38, and JNK signals and that these expressions were retained during inhibiting the proliferation of AGS cells. In other study for anti-cancer effect of CY, Park et al. demonstrated that CY induces the apoptosis through inhibiting the activation of PI3K/AKT signal pathway in A549 human lung carcinoma cells [9]. However, in present study, the phosphorylation of AKT were not inhibited by FCY in AGS cells. Additionally, in inhibitor study, the inhibition of MAPK signaling by specific protein inhibitors (ERK inhibitor: PD98059, p38 inhibitor: SB203580, and JNK inhibitor SP600125) protected cells from the cytotoxic effects of FCY whereas PI3K did not contribute to the anti-proliferative effect of FCY, which suggests that the activation of MAPK cascades play opposite roles in the proliferation of AGS cells.

The present results demonstrated that the fermentation of CY generates different chemical profiles than non-fermented CY (Fig. 1b). Bacterial fermentation either increases the levels of or generates active components that suppress tumor formation. The components that are structurally changed following bacterial fermentation include deglycosylates, sulfates, and flavonoids; these compounds increase the absorption rate of organs such as the liver and intestine [36], and may improve the bioactivity and bioavailability of the active components [37, 38]. In turn, these improvements may have beneficial effects during cancer therapy. In the present study, the fermentation of CY with *Lactobacillus delbrueckii* increased the content of active components, including ephedrine HCl, glycyrrhizin, 6-gingerol, schisandrin, and gomisin A, compared to unfermented CY. The extract of *E. sinica* contains ephedrine HCl, one of the constituents of CY, which has been shown to possess antiangiogenic, anti-invasive, and antitumor activities in B16F10 mouse melanoma cells [11]. Additionally, glycyrrhizin, which is isolated from *G. glabra*, has been shown to have anti-carcinogenic effects in human stomach cancer KATO III cells and promyelocytic leukemia HL-60 cells via the induction of caspase-dependent apoptosis [39]. Schisandrin, which is present in the fruit of *S. chinensis*, inhibits the growth of by arresting the cell cycle in the G1/G1 phase [40]. Gomisin A, which is isolated from *S. chinensis*, also exerts significant anti-carcinogenic effects. For example, gomisin A inhibits cell proliferation and arrests the cell cycle during the G1 phase in HeLa cells and induces apoptotic activity in HCT116 human colon cancer cells via the cleavage of caspase-7 [34, 41]. 6-Gingerol, which is the major active component of *Z. officinale*, induces caspase-3-dependent apoptosis and autophagy in HeLa cells, inhibits the growth of Lovo human colon cancer cells by arresting the cell cycle in the G2/M phase, and effectively suppresses tumor growth in a HCT116 cell xenograft assay via the inhibition of leukotriene A(4) hydrolase (LTA[4]H) [42–44].

Based on the above evidence, the changes in the various components of CY following bacterial fermentation may improve its anti-carcinogenic effects in the FCY formulation. In the present xenograft assays, FCY significantly suppressed the tumor growth of subcutaneously injected cancer cells compared to CY. Despite the enhancement of anti-carcinogenic effects following fermentation, there were no adverse side effects such as body weight loss, organ abnormalities, or changes in hematological and/or serological parameters (data not shown). Taken together, the present results demonstrate that the anti-carcinogenic effects of FCY may be enhanced compared to those of CY due to the alteration of its active components by bacterial fermentation.

## Conclusions

The present study assessed the anti-carcinogenic effects of FCY in vitro and in vivo. The findings strongly indicate that FCY induced apoptosis via the activation of caspases and the regulation of MAPK activity in AGS cells. Furthermore, the oral administration of FCY enhanced the inhibition of tumor formation, which suggests that bacterial fermentation improved the inhibitory effects of CY on cancer cells by changing the nature of its active components. Thus, it is suggested here that FCY may be useful as an herbal medicine for controlling malignant tumor growth. However, additional studies are required to identify the active components of FCY and their capabilities.
